# Nomogram to predict 5-year global cognitive impairment in *de novo* Parkinson disease with normal cognition at baseline

**DOI:** 10.3389/fnins.2025.1713488

**Published:** 2025-12-04

**Authors:** Xin Wang, Lu Tian

**Affiliations:** 1 Department of Neurosurgery, Shandong Provincial Hospital Affiliated to Shandong First Medical University, Jinan, Shandong, China; 2 Department of Neurosurgery, The First Affiliated Hospital of Shandong First Medical University & Shandong Provincial Qianfoshan Hospital, Jinan, Shandong, China; 3 Economic Operation Management Office, The First Affiliated Hospital of Shandong First Medical University & Shandong Provincial Qianfoshan Hospital, Jinan, Shandong, China

**Keywords:** Parkinson disease, predictive factors, global cognitive impairment, 5-yearfollow-up period, nomogram

## Abstract

**Background and objective:**

Cognitive impairment (CI, combing mild cognitive impairment and dementia) seriously affects the quality of life in patients with *de novo* Parkinson disease (PD). The aim of the present study was to identify the potential predictive factors for 5-year cognitive decline in *de novo* PD.

**Methods:**

Parkinson’s Progression Marker Initiative (PPMI) database was retrieved and PD patients with normal global cognition at baseline were included. These patients were divided into normal cognitive (NC) group and CI group based on their Montreal Cognitive Assessment (MoCA) scores at the 5-year follow-up period. A total of 66 baseline variables were compared between these two groups. Univariate and multivariate logistic regression analyses were conducted, followed by the development and validation of a nomogram to predict 5-year global cognitive decline in *de novo* PD patients.

**Results:**

A total of 344 PD patients with normal baseline cognition were included, in which 73 individuals developed CI at the 5 year follow-up period. Baseline MoCA, Benton Judgment of Line Orientation (BJOLO), Hopkins Verbal Learning Test (HVLT) immediate recall, Letter Number Sequencing (LNS), Symbol-Digit Modalities Test (SDMT), Semantic Fluency Test (SFT) scores, and Scale for Outcomes in Parkinson’s disease for Autonomic symptoms (SCOPA-AUT) total, gastrointestinal, and sexual dysfunction scores were selected out from the 66 potential predictors based on logistic regression analysis. These predictors were finally included in the nomogram of the model. The area under the ROC curve of the model was 0.870 (95% CI, 0.825–0.915).

**Conclusion:**

Our study constructed a model that predicts 5-year cognitive decline in *de novo* PD with high accuracy, which may allow for the early risk stratification of future CI in PD patients at baseline.

## 1 Introduction

Parkinson disease (PD), manifested by resting tremor, bradykinesia, rigidity, and imbalance, is the most common movement disorder (Bloem et al., 2021; Tanner and Ostrem, 2024). PD is also characterized by pathologies including misfolded *α*-synuclein in specific brain areas (Tanner and Ostrem, 2024). In addition to motor symptoms, patients often suffer from non-motor symptoms (NMS) including autonomic dysfunctions, depression, and cognitive function, some of which may appear at an earlier stage of the disease and progress with the duration of the disease (Bloem et al., 2021; Tanner and Ostrem, 2024).

Cognitive impairment (CI), combing mild cognitive impairment (MCI) and dementia, is one of the most common NMS in all stages of PD, with the prevalence rate as 6 times as in the normal populations at a similar age (Aarsland et al., 2001; Aarsland et al., 2021). As suggested by Chen et al. (2023), the critical difference between MCI and dementia depends on whether daily life function is severely affected: cognitive decline is not serious enough to disturb the daily activities in MCI patients (Litvan et al., 2012); whereas patients with PD-related dementia (PDD) suffered from CI severe enough to interfere their functional independence (Emre et al., 2007). It was reported that CI caused heavier burdens than all of the motor symptoms to the economic and healthcare system (Aarsland et al., 2021). Thus, risk estimation for cognitive decline at early stage of PD has high clinical significance.

Risk factors for future cognitive decline in PD was previously investigated in several original studies and reviews (Carlisle et al., 2023; Chen et al., 2023; Liu et al., 2017). The study of Chen et al. (2023) found that age, hypertension, baseline MoCA scores, Movement disorder society Unified PD Rating Scale part III (MDS-UPDRS III) scores, as well as apolipoprotein E (APOE) status were correlated to the development of future CI. In other studies, baseline general cognition, APOE status, CSF light ligament, freezing of gait, and dopamine deficit were associated with cognitive decline in PD individuals (Dijkstra et al., 2022; Qu et al., 2023; Ruiz Barrio et al., 2023). Overall, obvious methodological differences existed in these studies, and controversies still remain. Moreover, findings in the previous studies could not be directly generalized to the *de novo* PD patient with normal global cognition at baseline. Thus, it is imperative to construct a model specifically to predict long-term future CI in this patient group.

The present cohort study included *de novo* PD patients who had normal global cognition at baseline and investigated the potential predictors of future CI (combing MCI and PDD) in the 5-year follow-up visit. A robust model with good accuracy was to be developed, with the aim of allowing for early interventions for the individuals with high risk of future CI.

## 2 Materials and methods

### 2.1 Patient populations

Data was acquired from Parkinson’s Progression Markers Initiative (PPMI, www.ppmi-info.org) database in July, 2025. PPMI is an international and multicenter study involving *de novo* PD patients (diagnosed within 2 years) with dopamine transporter deficiencies confirmed by single-photon-emission CT, and written informed consent was acquired from each PPMI participant (Lang et al., 2023; Marek et al., 2018; Simuni et al., 2018). Based on the PPMI database, this 5-year cohort study included the de novo PD patients satisfying the following criteria: (1) had normal global cognition measured by MoCA scores at baseline; (2) with available MoCA data at the 5-year follow-up visit; (3) were followed-up annually up to 5 years. Patients with global cognitive impairment already at baseline were excluded from the study.

### 2.2 Outcome measures

The outcome of interests in this study is general cognitive decline (combing MCI and dementia) during the 5-year follow-up period. Consistent with the previous studies and guideline criteria, the cutoff value of <21/30 in MoCA score was to define dementia, and <26/30 was for MCI (Chen et al., 2023; Emre et al., 2007; Litvan et al., 2012). Apart from global cognition, other cognitive dimensions (memory, language, etc.) were evaluated using a series of specialized scales. For example, visuo-spatial function was measured with Benton Judgment of Line Orientation (BJOLO); and attention and processing performance was evaluated using Symbol-Digit Modalities Test (SDMT). Letter Number Sequencing (LNS) and Semantic Fluency Test (SFT) were used to assess the execution and working memory of the patients.

### 2.3 Data collection

A total of 66 potential clinical, radiological, and biomarker predictors were investigated. These variables were labeled as X1-X66. Clinical items included age at enrollment/onset (years), education (years), disease duration (years), sex (M/F), family history (yes/no), handedness, race (white/others), BMI, affected side and original symptoms including resting tremor, rigidity, bradykinesia, and postural instability (yes/no). Data regarding the overall severity of the disease included MDS-UPDRS part I-III, and the Hoehn and Yahr stage. Motor symptoms were evaluated with UPDRS-III, whereas autonomic disorders were measured with Scale for Outcomes in Parkinson’s disease for Autonomic symptoms (SCOPA-AUT). Epworth Sleepiness Scale and REM Sleep Behavior Disorder Screening Questionnaire (RBDSQ) were used to measure sleep quality and possible RBD, respectively. Other NMS were assessed with BJOLO, SDMT, LNS, and SFT for various cognitive domains, Geriatric Depression Scale (GDS) for depression, and State–Trait Anxiety Index (STAI) for anxiety. With regard to variables in radiology, we recorded DAT imaging data in various brain regions, and the putaminal or caudate asymmetry was calculated with the value in the side with the higher uptake divided by side with the lower uptake (Chen et al., 2023). Biomarker variables included *α* synuclein, APOE *ε4* status, serum uric acid, CSF α synuclein, Aβ42, neurofilament light, tau level, etc.

### 2.4 Statistical analysis

Patient were divided into normal cognition (NC) group and CI group based on 5-year cognitive performance. Each baseline variables were compared separately between the two groups. Two-sampled *t*-test was used for continuous variables with normal distributions, Mann–Whitney test for non-normal distributions, and categorical variables were compared using *χ*^2^ tests. Missing values were handled with mean interpolation (Emmanuel et al., 2021). In the next step, variables with value of *p* < 0.1 were included in the univariate logistic regression model, followed by the inclusion of the variables with value of *p* < 0.05 and without obvious inter-individual correlations (*r* ˃ 0.5) into the multivariate regression analyses. Inter-variable correlations were assessed with *Spearman* or *Pearson* correlation analysis, based on the distribution characteristics of the data. If inter-individual correlation was found, the variable with a lower *p* value was selected. In the multivariate regression model, “backward LR method” was used to identify the final significant predictors (*p* < 0.05). The bootstrap resampling method (*B* = 1,000 repetitions) was used for internal verification of the model. A nomogram was constructed, followed by the plotting of ROC curve, and calculation of C statistic for model accuracy evaluation. All statistical analyses were conducted with R software version 4.4.3 (R Foundation for Statistical Computing), and the value of *p*< 0.05 was defined as statistically significant.

## 3 Results

**Figure 1:**
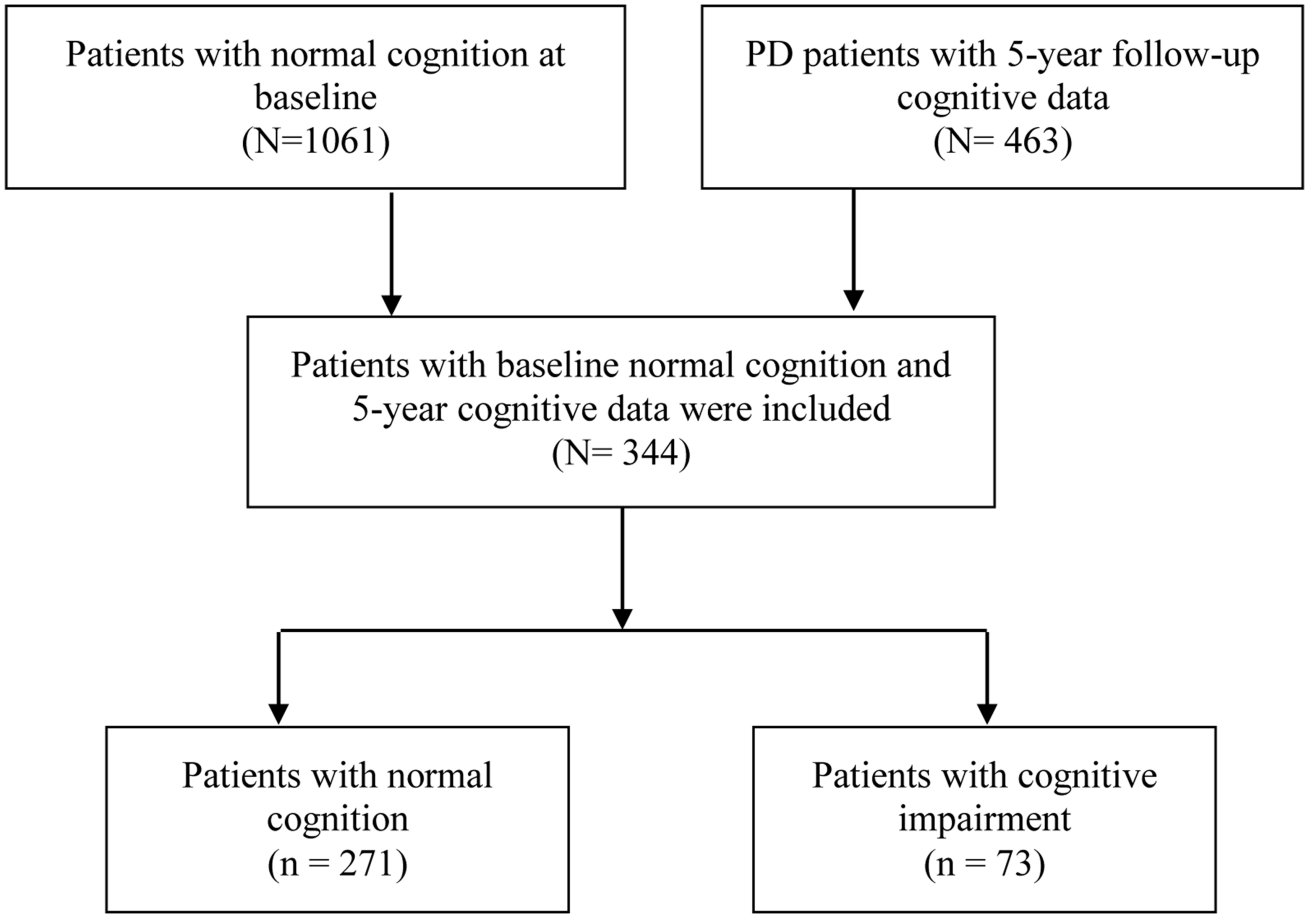
Selection, enrollment and classification of the patients.

The patient selection procedure was shown in Figure 1. A total of 344 *de novo* PD patients with normal cognitive function at baseline and 5-year cognitive data available were included. At the 5-year follow-up visit, 271 patients were divided into normal cognition group, whereas other 73 subjects were classified as “cognitive impairment” based on the MoCA scores.

Results in comparisons regarding continuous variables were shown in Table 1. Subjects in CI group had significantly higher age at onset and lower education levels than patients in NC group. Besides, MoCA, BJOLO, HVLT, LNS, SDMT, and SFT scores were significantly lower in CI patient group than NC group (*p* < 0.001). Moreover, the RBDSQ scores in CI patient group were significantly higher than NC group (4.97 [3.11] vs. 3.77 [2.49]; *p* = 0.004), indicating more severe sleep disturbance in CI patient group at baseline. Significant between-group differences also existed in the STAI State Sub-score, SCOPA-AUT total and gastrointestinal sub-score, as well as MDS-UPDRS I-III score (*p* < 0.05). With regard to biomarker and radiological variables, putaminal asymmetry was the only variable with significant between-group difference. As is shown in Table 2, with regard to binary/categorical factors, significant between-group difference only existed in the variable of sex (*p* = 0.02). These analyses were repeated through imputing missing data with means, and the missing data did not obviously change the results in our analyses (data not shown).

**Table 1:** Comparisons of the continuous variables between the two groups.

Baseline characteristics	Normal cognition group	Cognitive impairment group	*p*-value
X1 Age (enrollment), years	58.88 (9.68)	64.52 (8.28)	**0.000**
X2 Education, years	15.91 (3.02)	15.08 (3.46)	**0.000**
X3 BMI	26.43 (4.37)	27.55 (5.28)	0.203
X4 Age (onset of disease), years	55.92 (9.84)	61.81 (8.71)	**0.000**
X5 Disease duration, years	1.46 (1.86)	1.20 (1.61)	0.505
X6 Baseline MoCA score	28.20 (1.29)	27.36 (1.16)	**0.000**
X7 Benton Judgement of Line Orientation Score	13.31 (2.51)	11.66 (3.19)	**0.000**
X8 HVLT Discrimination Recognition Index	10.45 (1.86)	9.21 (2.63)	**0.000**
X9 HVLT Immediate Recall	26.61 (4.19)	22.34 (4.21)	**0.000**
X10 HVLT Delayed Recall	9.42 (2.09)	7.52 (2.13)	**0.000**
X11 HVLT Delayed Recognition	11.46 (0.85)	10.97 (1.13)	**0.000**
X12 Letter Number Sequencing Score	11.35 (2.53)	9.16 (2.43)	**0.000**
X13 Symbol Digit Modalities Test Score	44.64 (9.18)	36.40 (8.93)	**0.000**
X14 Semantic Fluency Test Score	22.47 (5.16)	19.10 (5.39)	**0.000**
X15 Epworth Sleepiness Scale Score	6.04 (3.84)	6.36 (3.75)	0.587
X16 REM Sleep Behavior Disorder Screening Questionnaire	3.77 (2.49)	4.97 (3.11)	**0.004**
X17 Geriatric Depression Scale Score	2.37 (2.62)	2.66 (2.40)	0.095
X18 State–Trait Anxiety Index (STAI) Total Score	65.42 (18.87)	67.95 (17.82)	0.178
X19 STAI State Sub-Score	32.59 (10.13)	35.36 (10.26)	**0.028**
X20 STAI Trait Sub-Score	32.82 (10.01)	32.59 (8.60)	0.728
X21 SCOPA-AUT Total Score	9.43 (6.64)	12.19 (7.92)	**0.003**
X22 SCOPA-AUT Gastrointestinal (GI) Sub-Score	1.98 (2.12)	3.26 (2.77)	**0.000**
X23 SCOPA-AUT Urinary Sub-Score	4.24 (3.15)	5.01 (3.41)	0.080
X24 SCOPA-AUT Cardiovascular Sub-Score	0.47 (0.77)	0.71 (1.14)	0.050
X25 SCOPA-AUT Thermoregulatory Sub-Score	1.39 (1.82)	1.26 (1.64)	0.820
X26 SCOPA-AUT Pupillomotor Sub-Score	0.38 (0.68)	0.49 (0.73)	0.144
X27 SCOPA-AUT Sexual Dysfunction Sub-Score	0.99 (1.45)	1.45 (1.88)	0.058
X28 MDS-UPDRS Part I Score OFF	5.35 (4.14)	6.85 (4.87)	**0.017**
X29 MDS-UPDRS Part II Score OFF	5.61 (4.26)	7.16 (4.85)	**0.006**
X30 MDS-UPDRS Part III Score OFF	19.23 (8.91)	22.85 (9.48)	**0.004**
X31 MDS-UPDRS Total Score OFF	30.16 (13.54)	36.01 (14.49)	**0.002**
X32 CSF A-beta 1–42	835.12 (293.99)	801.04 (290.75)	0.409
X33 CSF t-tau	166.04 (53.25)	155.39 (44.57)	0.074
X34 CSF p-tau	14.42 (4.72)	13.35 (4.18)	0.088
X35 CSF alpha-synuclein	1497.46 (651.18)	1330.04 (490.00)	0.107
X36 serum uric acid (mg/dL)	5.14 (1.32)	5.46 (1.43)	0.127
X37 CSF Neurofilament Light	88.35 (42.92)	107.27 (59.94)	0.092
X38 DaTscan left caudate	1.98 (0.57)	1.90 (0.65)	0.322
X39 DaTscan right caudate	1.97 (0.56)	1.91 (0.68)	0.449
X40 Mean caudate measure	1.97 (0.51)	1.90 (0.64)	0.344
X41 DaTscan left putamen	0.80 (0.34)	0.76 (0.36)	0.273
X42 DaTscan right putamen	0.82 (0.33)	0.81 (0.39)	0.468
X43 Mean putamen measure	0.81 (0.27)	0.79 (0.34)	0.171
X44 Mean striatum measure	1.39 (0.37)	1.35 (0.47)	0.391
X45 Aβ42:t-tau ratio	5.54 (1.36)	5.46 (1.73)	0.682
X46 Putaminal asymmetry	1.53 (0.46)	1.41 (0.41)	**0.015**
X47 Caudate asymmetry	1.23 (0.17)	1.22 (0.22)	0.119

Boldface type indicates statistical significance.

**Table 2:** Comparisons of the binary/categorical variables between the two groups.

Baseline characteristics	Patients without CI	Patients with CI	*p*-value
X48 α synuclein (yes/no)	225/28	65/4	0.195
X49 Sex (M/F)	156/115	53/20	**0.02**
X50 Race (white, others)	265/6	68/5	0.06
X51 Family history (yes/no)	95/176	25/48	0.898
X52 Handedness (right/left or mixed)	239/32	62/11	0.455
X53 Side of symptoms (left/right/symmetry)	122/144/5	31/39/3	0.517
X54 Resting tremor at diagnosis (yes/no)	195/75	58/14	0.152
X55 Rigidity at diagnosis (yes/no)	201/63	58/13	0.321
X56 Bradykinesia at diagnosis (yes/no)	222/45	63/9	0.37
X57 Postural Instability at diagnosis (yes/no)	21/246	11/62	0.062
X58 Impulsive-Compulsive disorders (yes/no)	75/196	17/55	0.489
X59 Orthostasis (yes/no)	34/234	10/63	0.819
X60 Hoehn & Yahr stage (stage 1/stage 2)	116/135	26/41	0.278
X61 TD/PIGD classification (TD/non-TD)	166/85	47/20	0.535
X62 Apathy (yes/no)	46/255	13/60	0.595
X63 Fatigue (yes/no)	136/135	42/31	0.265
X64 CSF SAA combined results (negative/positive)	24/228	3/64	0.187
X65 APOE ε4 status (negative/heterozygous/ homozygous)	210/56/5	55/15/3	0.532
X66 MDS-UPDRS-I Hallucinations and Psychosis (yes/no)	9/262	6/67	0.099

Boldface type indicates statistical significance.

Variables with values of *p* < 0.1 were included into the univariate logistic regression model. As is indicated in Table 3, the following variables were significantly associated with CI: age at enrollment, education, age at onset, baseline MoCA, BJOLO, HVLT discrimination recognition index, immediate recall, delayed recall, and relayed recognition, LNS scores, SDMT scores, RBDSQ scores, STAI State Sub-score, SCOPA-AUT total, gastrointestinal, cardiovascular, and sexual dysfunction sub-score, MDS-UPDRS Part I-III and total score, sex (M/F), and MDS-UPDRS-I hallucinations and psychosis (yes/no).

**Table 3:** Univariate logistic regression analyses regarding risk factors for cognitive impairment.

Clinical factors	Coefficient	COR (95% CI)	*p*-value
X1 Age (enrollment), years	0.07	1.073 (1.039–1.108)	**0.000**
X2 Education, years	−0.079	0.924 (0.854–0.999)	**0.048**
X4 Age (onset of disease), years	0.07	1.072 (1.039–1.107)	**0.000**
X6 Baseline MoCA score	−0.536	0.585 (0.468–0.731)	**0.000**
X7 Benton Judgement of Line Orientation Score	−0.205	0.814 (0.743–0.893)	**0.000**
X8 HVLT Discrimination Recognition Index	−0.236	0.790 (0.703–0.888)	**0.000**
X9 HVLT Immediate Recall	−0.236	0.790 (0.735–0.848)	**0.000**
X10 HVLT Delayed Recall	−0.379	0.684 (0.602–0.778)	**0.000**
X11 HVLT Delayed Recognition	−0.481	0.618 (0.479–0.798)	**0.000**
X12 Letter Number Sequencing Score	−0.365	0.694 (0.615–0.785)	**0.000**
X13 Symbol Digit Modalities Test Score	−1.000	0.905 (0.876–0.935)	**0.000**
X14 Semantic Fluency Test Score	−0.128	0.880 (0.833–0.929)	**0.000**
X16 REM Sleep Behavior Disorder Screening Questionnaire	0.157	1.170 (1.067–1.283)	**0.001**
X17 Geriatric Depression Scale Score	0.042	1.042 (0.947–1.148)	0.396
X19 STAI State Sub-score	0.026	1.026 (1.001–1.051)	**0.041**
X21 SCOPA-AUT Total Score	0.051	1.052 (1.017–1.089)	**0.004**
X22 SCOPA-AUT Gastrointestinal (GI) Sub-score	0.212	1.237 (1.114–1.373)	**0.000**
X23 SCOPA-AUT Urinary Sub-score	0.070	1.072 (0.994–1.157)	0.071
X24 SCOPA-AUT Cardiovascular Sub-score	0.289	1.335 (1.018–1.750)	**0.037**
X27 SCOPA-AUT Sexual Dysfunction Sub-score	0.172	1.187 (1.020–1.382)	**0.027**
X28 MDS-UPDRS Part I Score OFF	0.074	1.076 (1.018–1.138)	**0.010**
X29 MDS-UPDRS Part II Score OFF	0.074	1.077 (1.019–1.138)	**0.009**
X30 MDS-UPDRS Part III Score OFF	0.041	1.042 (1.013–1.072)	**0.005**
X31 MDS-UPDRS Total Score OFF	0.029	1.029 (1.010–1.048)	**0.003**
X34 CSF p-tau	−0.056	0.946 (0.885–1.010)	0.097
X36 Serum Uric Acid (mg/dL)	0.177	1.194 (0.982–1.452)	0.075
X37 CSF Neurofilament Light	0.007	1.008 (0.999–1.016)	0.096
X46 Putaminal asymmetry	−0.670	0.512 (0.257–1.018)	0.056
X49 Sex (M/F)	0.670	1.954 (1.107–3.447)	**0.021**
X50 Race (white, others)	1.178	3.248 (0.962–10.960)	0.058
X57 Postural Instability at diagnosis (yes/no)	0.732	2.078 (0.952–4.538)	0.066
X66 MDS-UPDRS-I Hallucinations and Psychosis (yes/no)	1.021	2.777 (1.119–6.888)	**0.028**

Boldface type indicates statistical significance.

**Figure 2:**
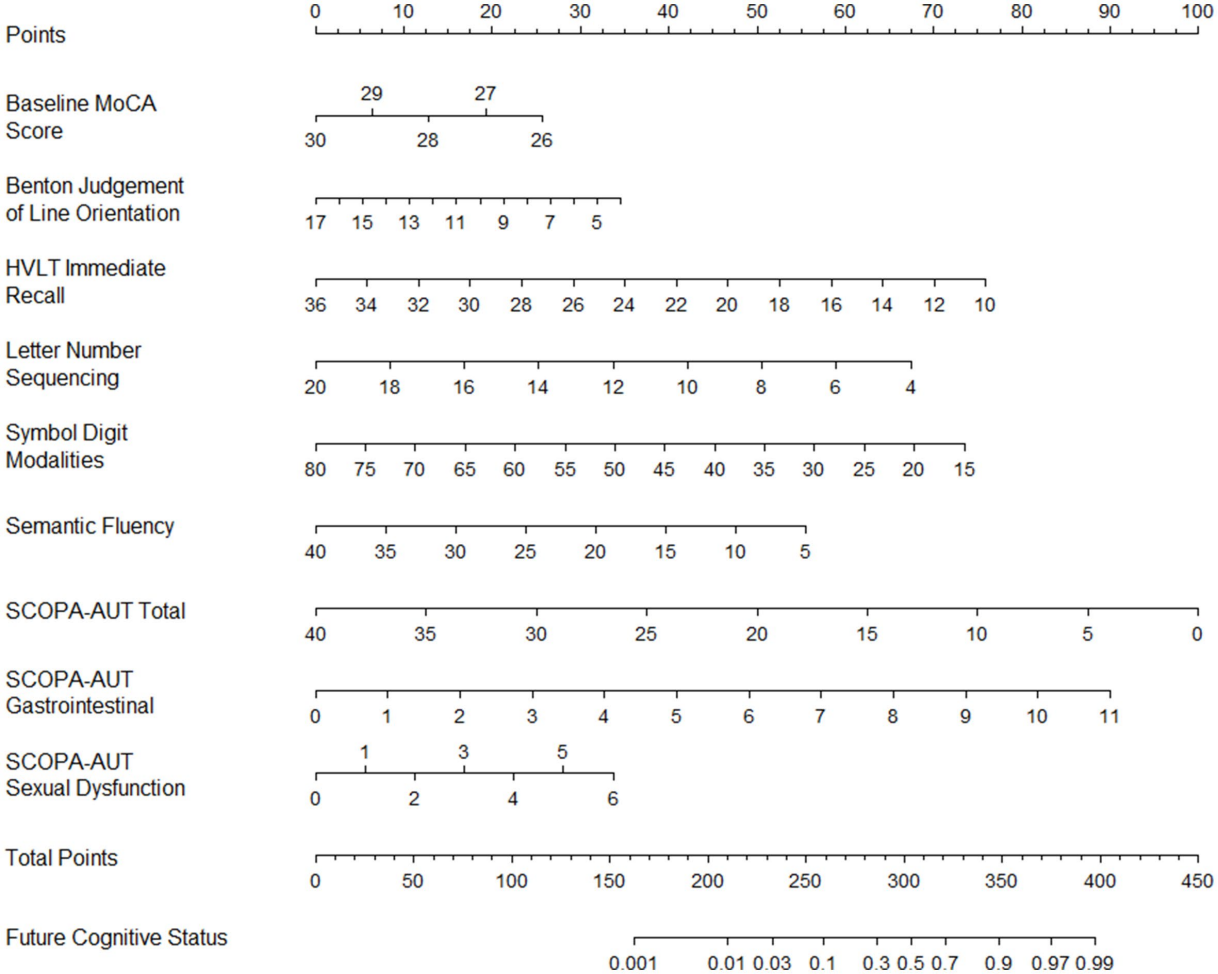
Nomogram for the risk of 5-year cognitive impairment in *de novo* PD patients with normal cognitive function at baseline.

**Figure 3:**
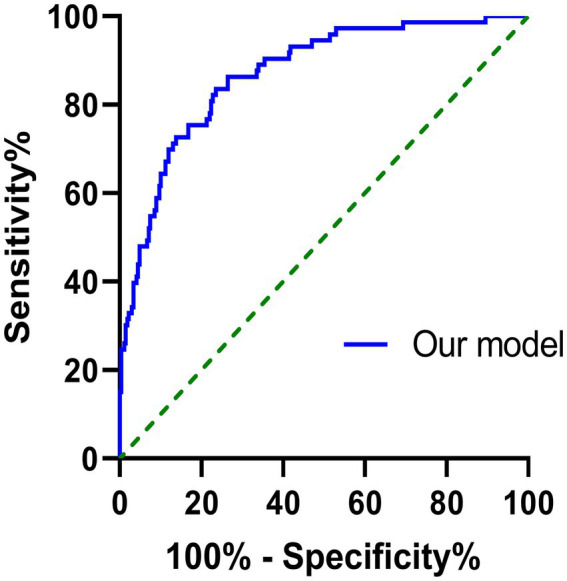
ROC curve for CI prediction at the 5-year follow-up visit in de novo PD patients with normal cognitive function at baseline.

**Figure 4:**
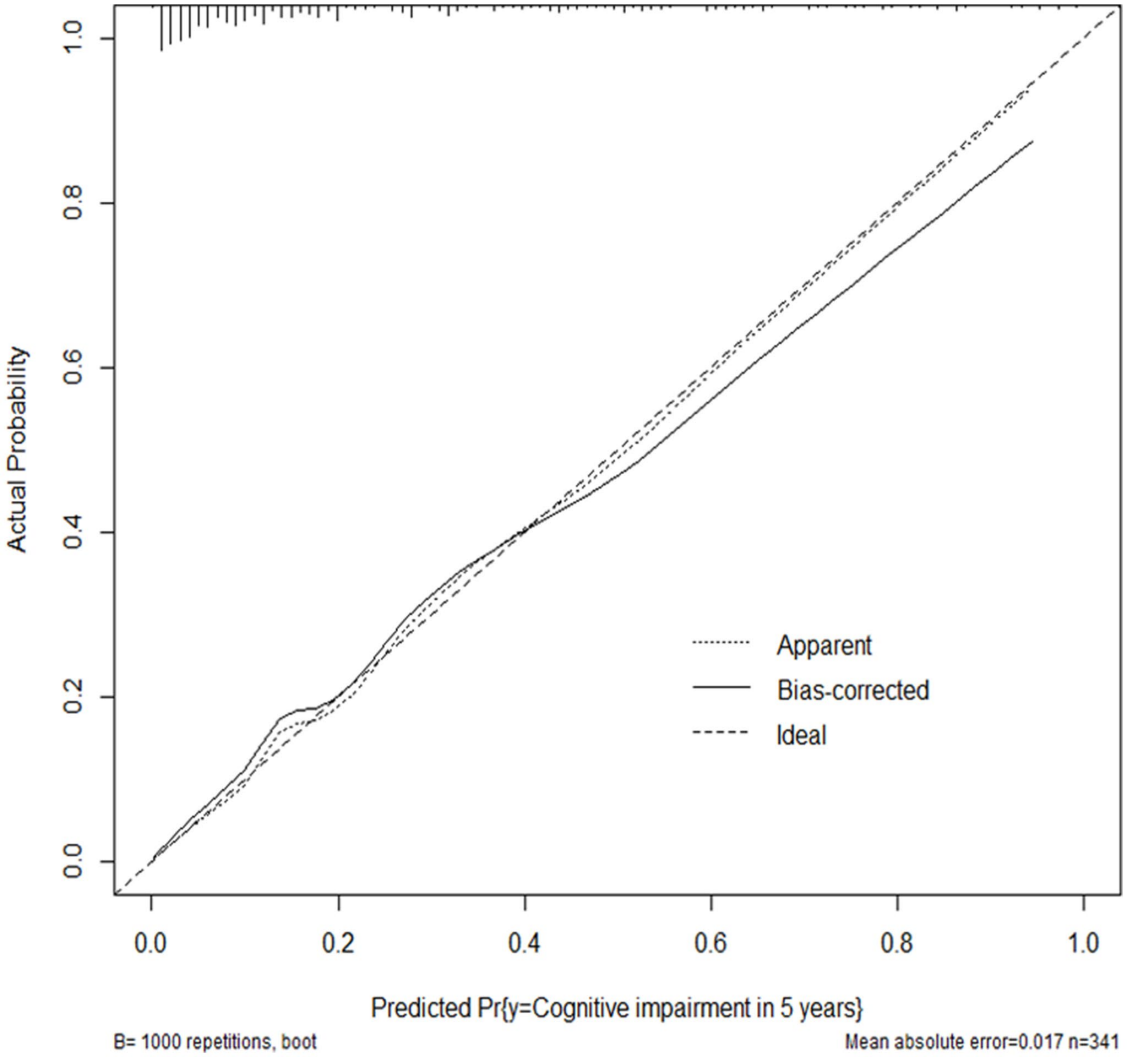
Calibration plot for the nomogram. The apparent and bias corrected values are close to each other, indicating that the nomogram has high predictive performance.

As is shown in Table 4, the multivariate regression model indicated that the baseline MoCA, BJOLO, HVLT immediate recall, LNS, SDMT, and SFT score, as well as SCOPA-AUT total, gastrointestinal, and sexual dysfunction sub-score were significantly correlated to the development of cognitive decline at 5-year follow-up period (p < 0.05), and they were finally included into the nomogram (Figure 2). C-statistics was 0.870. After the model was constructed, ROC curve of the model was drawn, and bootstrap resampling method (B = 1,000) from the original data set was performed to validate the model internally. As is shown in Figure 3, the area under the ROC curve of the model was 0.870 (95% CI, 0.825–0.915). In the calibration curve of our model (Figure 4), the linear regression slope between the predicted probability and the actual value was close to 1. These results indicated that our model could predict 5-year cognitive decline with good accuracy. Finally, comparisons were made between our final model and the simple model which only included age at enrollment, and the results also indicated that the final model was more reliable (Supplementary Figures S1–S3).

**Table 4:** Multivariate logistic regression analyses regarding risk factors for cognitive impairment.

Clinical factors	Coefficient	COR (95% CI)	*p*-value
X6 Baseline MoCA score	−0.315	0.730 (0.560–0.952)	**0.020**
X7 Benton Judgement of Line Orientation Score	−0.130	0.878 (0.780–0.988)	**0.030**
X9 HVLT Immediate Recall	−0.143	0.867 (0.800–0.939)	**0.000**
X12 Letter Number Sequencing Score	−0.206	0.814 (0.698–0.949)	**0.009**
X13 Symbol Digit Modalities Test Score	−0.055	0.946 (0.910–0.983)	**0.005**
X14 Semantic Fluency Test Score	−0.078	0.925 (0.867–0.988)	**0.020**
X21 SCOPA-AUT Total Score	−0.122	0.885 (0.808–0.969)	**0.008**
X22 SCOPA-AUT Gastrointestinal (GI) Sub-score	0.401	1.493 (1.169–1.907)	**0.001**
X27 SCOPA-AUT Sexual Dysfunction Sub-score	0.275	1.317 (1.042–1.663)	**0.021**
Constant	18.493	44608.324	**0.000**

Boldface type indicates statistical significance.

## 4 Discussion

Based on the high-quality data from PPMI database, we constructed a predictive model for future long-term cognitive decline in *de novo* PD patients with normal cognition at baseline. The patients were evaluated comprehensively with a variety of standardized neuropsychological tests annually for up to 5 years. A total of 66 baseline potential predictors were analyzed in our study. Using our model combing baseline MoCA, BJOLO, HVLT immediate recall, LNS, SDMT, SFT, and SCOPA-AUT total, gastrointestinal, and sexual dysfunction scores, the 5-year CI could be predicted with high accuracy. The area under the ROC curve of the model was 0.870 (95% CI, 0.825–0.915). All of the related evaluations could be conducted in clinic, indicating the high feasibility of our model in real practice. To the best of our knowledge, this is the first study to construct a nomogram for predicting cognitive decline in a long period up to 5 years. Our results allow for identifying individuals with high risk for future CI, holding the promise to facilitate the intervention of PD-related CI at an early stage.

As mentioned earlier, several previous publications (Baiano et al., 2020; Carlisle et al., 2023; Chen et al., 2023; Guo et al., 2019; Liu et al., 2017) attempted to identify the relevant factors for PD-related cognitive decline, but consensuses are far from being established. Age was recognized as an independent predictive factor for CI in PD patients in many studies. For example, a recent systematic review indicated that advanced age, later disease onset, longer disease duration, as well as greater disease severity are critical risk factors for development of MCI in PD (Zhang et al., 2025). In the study of Liu et al. (2017), age at onset, baseline MMSE, education, motor score, sexual function, depression, as well as β-glucocerebrosidase (GBA) mutation status were included in the final prediction model of cognitive status in PD. In addition, the study of Baiano et al. (2020) suggested that the development of MCI in PD patients was associated with higher age, shorter education time, longer disease duration, increased levodopa equivalent daily dose (LEDD), and more serious motor manifestations. Guo et al. (2019) indicated that the following 9 modifiable factors might increase the risk of cognitive decline in PD patients: postural-instability-gait disorder, hallucinations, orthostatic hypotension, cerebrovascular disease, diabetes mellitus, obesity, cardiac disease, alcohol consumption, and smoking. Another study (Carlisle et al., 2023) suggested that age, hallucination, excessive daytime sleepiness, and motor symptoms were associated with cognitive decline in PD. Besides, Chen et al. (2023) suggested that predictive accuracy of CI based on age alone could be improved by the addition of concurrent hypertension, baseline MoCA and MDS-UPDRS III scores, as well as APOE status (AUC 0.80 [95% CI 0.74–0.86] vs. 0.71 [0.64–0.77], *p* = 0.008). However, older age was not a significant predictors in our model. On the whole, this inconsistency needed to be interpreted with caution. We thought that cognitive performance may decline physiologically with age in general population, but older age itself may not necessarily be an independent predictor of CI in PD patients.

Consistent to the study of Chen et al. (2023), baseline MoCA score was also recognized as an independent predictive factor for CI in PD patients. Patients in the CI group had decreased MoCA scores than subjects in NC group at baseline (27.36 [1.16] vs. 28.20 [1.29]). Besides, in the study of Liu et al. (2017) baseline global cognition assessed with MMSE was also included in the prediction of cognition in PD. These results suggested that subtle differences in global cognition already existed prior to the development of symptomatic cognitive decline. Our logistic regression analysis suggested that baseline MoCA score was a protective factor for future CI, and with 1 point increase in MoCA score at baseline, the risk of 5-year cognitive decline will decrease by 27.0%. Better performance in MoCA test indicates less pathological lesions underlying PD, thus it is associated with reduced risk of cognitive decline.

Apart from general cognitive function, significant between-group differences were also found in various cognitive subdomains at baseline, such as visuo-spatial function measured by BJOLO and working memory measured by SFT. This phenomena probably indicated that the development of symptomatic global cognitive decline lagged far behind specific cognitive subdomains in an extent. In these variables, baseline BJOLO score for visuo-spatial function, HVLT immediate recall score for language learning, LNS score for execution, SDMT score for attention and processing performance, as well as SFT score for working memory were independent predictive factors for 5-year CI in PD. This result suggested that although the subjects all appeared to have normal global cognition at baseline, deficits in cognitive subdomains may appear earlier in the patients with high risks for future symptomatic cognitive decline. Thus it is imperative to comprehensively assess different cognitive subdomains for PD at an early stage.

In addition to cognitive measures, SCOPA-AUT scores for autonomic dysfunction were also suggested to be independent predictive factors for future CI. This finding was in accordance to a latest meta-analytic study (Wang et al., 2025) which suggested that constipation might significantly increase the risk of cognitive decline, especially in PD patient group. Disorders of autonomic function including symptomatic gastrointestinal motility dysfunction and sexual dysfunction, are common, usually preceding motor symptoms by years and deteriorating with the duration of the disease (Montanari et al., 2023; Tanner and Ostrem, 2024). As for the sexual dysfunction, the study of Liu et al. (2017) also indicated that sexual function was a significant factor in the prediction model of cognition in PD. With regard to the intestinal functions, the connections between disease progression in the gut and subsequent dysfunctions in the brain was not totally understood (Bloem et al., 2021). One study suggested that alterations in gut microbiota and increased intestinal permeability may result in the development of PD (Bhattarai et al., 2021). Another study suggested that constipation occurs earlier than the appearance of motor symptoms by years (Postuma et al., 2019), which was in accordance with the Braak hypothesis that PD is triggered when foreign substances invaded the central nervous system, probably via the gastrointestinal system, spreading via the vagal nerve into the brain (Bloem et al., 2021; Braak et al., 2003). On the other hand, bacterial infection in the gut lead to mitochondrial antigen presentation and results in autoimmune mechanisms in mice (Matheoud et al., 2019). In addition, mice overexpressing SNCA develop parkinsonism and cerebral dysfunctions only in the presence of gut germ, with microbiota-free mice protecting against neuro-degeneration (Sampson et al., 2016). On the whole, further investigations are needed to evaluate whether alterations in the microbiome have any causal connections to PD-related CI or whether the microbiome only reflect secondary changes. Our study suggested the necessities in early assessment of autonomic dysfunction, which may also allow for appropriate interventions at an early stage.

With regard to radiological variables and biomarker items, no item was finally recognized as an independent predictive factor of future CI in PD patients. Although putaminal asymmetry was the only variable with significant between-group difference (1.53 [0.46] vs. 1.41 [0.41], *p* = 0.015), it was not included into the final model by the multivariate regression analysis. Compare with PD patients without cognitive decline, PD patients with CI appeared to have more widespread denervation of dopaminergic terminals in basal nuclei (Sasikumar and Strafella, 2020). Thus pathology in the putaminal nucleus could disturb one’s normal cognition in theory. However, in the perspective of brain network, putaminal nucleus mainly involves in specific cognitive subdomains such as stimulus–response, or habit, learning, instead of global cognition (Grahn et al., 2008). This may partly interpret the absence of putaminal asymmetry in the final predictive model. As for the biological factors, some studies indicated that alterations in amyloid-β1–42 (Aβ42) or total *α*-synuclein levels in CSF were related to CI in patients with PD (Aarsland et al., 2021; Lin and Wu, 2015; Schrag et al., 2017), whereas other study found no associations (Chen et al., 2023). A recent systematic review suggested a significant association between decreased serum BDNF levels and cognitive decline in PD (Zhao et al., 2025). However, our study suggested no significant predictive value of biomarker variables in the development of cognitive decline. Overall, the exact value of putaminal nucleus and biomarkers in the pathogenesis of PD-related cognitive function warrants further research in the future.

As mentioned earlier, NMS were recognized as heavy burdens in PD, and these symptoms sometimes occur before the diagnosis and almost inevitably emerge with the whole course of the disease (Chaudhuri et al., 2006; Sauerbier et al., 2016). NMS even dominate the clinical picture of advanced Parkinson’s disease and result in serious disability, poor quality of life, as well as shortened life expectancy (Chaudhuri et al., 2006). Our study indicated that some of the NMS which could be improved with available treatments (such as constipation, and genitourinary problems) were correlated to the long-term cognitive decline. Therefore attention being focused on the recognition and treatment of these NMS, holds the promise to slowing or preventing the progression of cognitive decline and facilitating the management of PD in the long-term.

Several limitations need to be mentioned. Firstly, CI group was not further subdivided into MCI and PDD group, due to the small sample size, and the conversion from MCI to dementia was not analyzed either. Secondly, we mainly evaluated the predictive factors of long-term global cognition in PD patients, predictors of other cognitive domains such as working memory and execution need to be assessed in future studies. Thirdly, there is a lack of external validation in our study, PPMI is an international and multicenter study conducted mostly on western developed countries, thus the performance and robustness of our model probably needed to be further tested in external validation sets consisting of different PD patient populations such as from Asia. Fourthly, MoCA alone was used as the outcome measure in our study, and other outcome scales such as MMSE needed to be evaluated in the future studies. Besides, the sample size was small, relative to the number of factors included in our predictive model, potentially indicating overfitting. Thus, our model needed to be validated in future studies. Finally, predictor factors identified in our study mainly include semi-objective cognitive rating score and autonomic symptoms, lacking effective biomarkers, which may limit the predictive efficacy in an extent. Therefore, large studies are warranted in the future.

## 5 Conclusion

Using our nomogram model combing baseline MoCA, BJOLO, HVLT immediate recall, LNS, SDMT, SFT score, and SCOPA-AUT total, gastrointestinal, and sexual dysfunction scores, the cognitive decline at a long stage up to 5 years could be predicted with high accuracy. This model need to be further validated in a larger external sample in the future studies.

## Data Availability

The raw data supporting the conclusions of this article will be made available by the authors, without undue reservation.

## Supplementary material

SUPPLEMENTARY FIGURE S1 Nomogram decision curve (DCA) for the risk of the cognitive impairment. The simple model (red curve) included the age at enrollment, and the complex model (blue curve) is our final model, this plot indicated that compared with the simple model, our final model could bring large extent of net benefit.

SUPPLEMENTARY FIGURE S2 Clinical impact analysis of the nomogram (simple model). The red curve (number of high risk) indicates the number of people classified as positive (high risk) by the nomogram for each threshold probability. The blue curve (number of high risk with the outcome) represents the number of true positive under each threshold probability.

SUPPLEMENTARY FIGURE S3 Clinical impact analysis of the nomogram (complex model). The red curve (number of high risk) indicates the number of people classified as positive (high risk) by the nomogram for each threshold probability. The blue curve (number of high risk with the outcome) represents the number of true positive under each threshold probability.
