# Supplementary figures and images for: Nomogram to predict 5-year global cognitive impairment in de novo Parkinson disease with normal cognition at baseline
Source: Front Neurosci. 2025 Dec 4;19:1713488. doi: 10.3389/fnins.2025.1713488 (PMC12711850; doi:10.3389/fnins.2025.1713488)

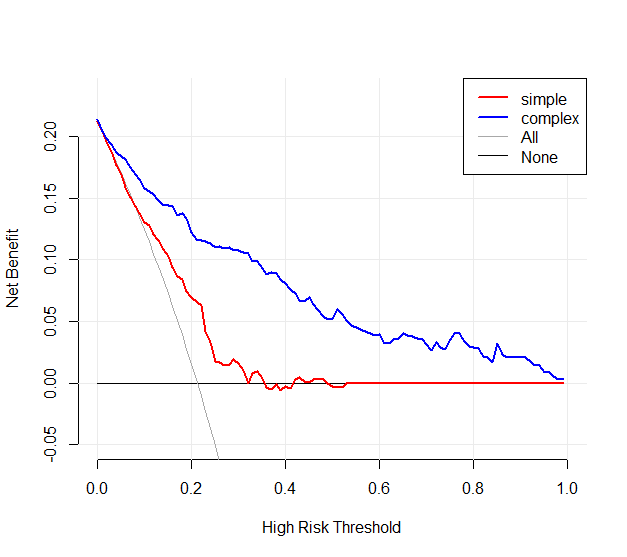

Supplement: SUPPLEMENTARY FIGURE S1 — Nomogram decision curve (DCA) for the risk of the cognitive impairment. The simple model (red curve) included the age at enrollment, and the complex model (blue curve) is our final model, this plot indicated that compared with the simple model, our final model could bring large extent of net benefit. [file Image_1.TIFF]

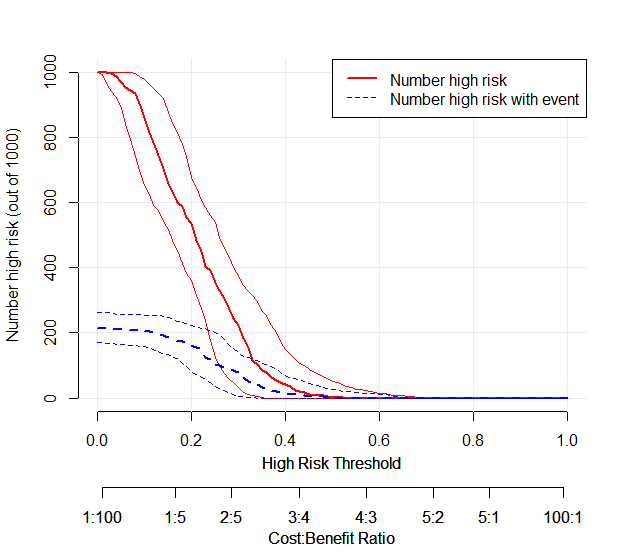

Supplement: SUPPLEMENTARY FIGURE S2 — Clinical impact analysis of the nomogram (simple model). The red curve (number of high risk) indicates the number of people classified as positive (high risk) by the nomogram for each threshold probability. The blue curve (number of high risk with the outcome) represents the number of true positive under each threshold probability. [file Image_2.TIFF]

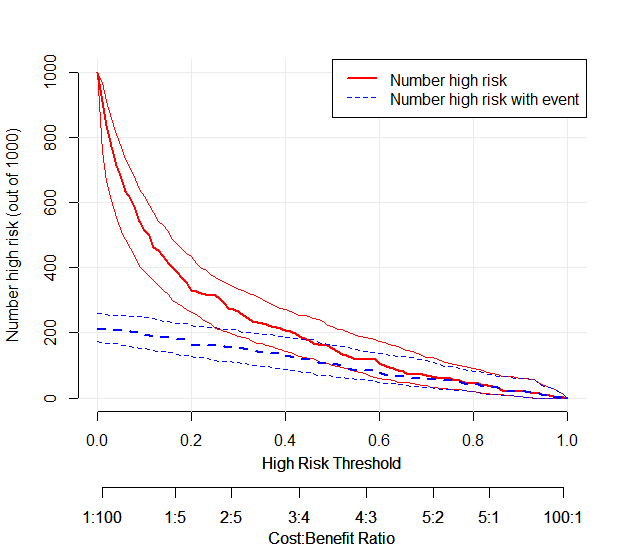

Supplement: SUPPLEMENTARY FIGURE S3 — Clinical impact analysis of the nomogram (complex model). The red curve (number of high risk) indicates the number of people classified as positive (high risk) by the nomogram for each threshold probability. The blue curve (number of high risk with the outcome) represents the number of true positive under each threshold probability. [file Image_3.TIFF]
